# 

**Published:** 1915-12

**Authors:** 


DECEMBER, 1915. SPECIAL NUMBER.

*Vol.* XXXIII. *No.* 129.

### THE BRISTOL MEDICO-CHIRURGICAL JOURNAL

*PUBLISHED UNDER THE AUSPICES OF*

*THE BRISTOL MEDICO-CHIRURGICAL SOCIETY.*

*Editor:* P. WATSON-WILLIAMS, M.D.

WITH WHOM ARE ASSOCIATED

J. MICHELL CLARKE, M.A., M.D., LL.D.,

J. LACY FIRTH, M.S., M.D., JAMES SWAIN, M.S., M.D.

J. A. NIXON, B.A., M.D., *Assistant Editor.*

*Editorial Secretary:* J. M. FORTESCUE-BRICKDALE, M.A., M.D.

BRISTOL: J. W. ARROWSMITH LTD.

LONDON: J. & A. CHURCHILL, 7 GREAT MARLBOROUGH STREET.

*Price Two Shillings and Sixpence.*

